# Zinc-Phthalocyanine-Loaded Extracellular Vesicles Increase Efficacy and Selectivity of Photodynamic Therapy in Co-Culture and Preclinical Models of Colon Cancer

**DOI:** 10.3390/pharmaceutics13101547

**Published:** 2021-09-23

**Authors:** Pablo Lara, Ruben V. Huis in ‘t Veld, Carla Jorquera-Cordero, Alan B. Chan, Ferry Ossendorp, Luis J. Cruz

**Affiliations:** 1 Percuros B.V., 2333 CL Leiden, The Netherlands; p.lara_arenas@lumc.nl (P.L.); C.A.Jorquera@lumc.nl (C.J.-C.); alanchan@clara.net (A.B.C.); 2 Translational Nanobiomaterials and Imaging (TNI) Group, Radiology Department, Leiden University Medical Center, 2333 ZA Leiden, The Netherlands; R.V.Huis_in_t_Veld@lumc.nl; 3 Department of Orthopedics, University Medical Center Utrecht, 3584 CX Utrecht, The Netherlands; 4 Department of Immunology, LUMC, 2333 ZA Leiden, The Netherlands; F.A.Ossendorp@lumc.nl

**Keywords:** extracellular vesicles (EVs), exosomes, targeting, tracking, drug delivery, theranostics, photodynamic therapy, PDT, zinc phthalocyanine

## Abstract

Photodynamic therapy (PDT) is a promising and clinically approved method for the treatment of cancer. However, the efficacy of PDT is often limited by the poor selectivity and distribution of the photosensitizers (PS) toward the malignant tumors, resulting in prolonged periods of skin photosensitivity. In this work, we present a simple and straightforward strategy to increase the tumor distribution, selectivity, and efficacy of lipophilic PS zinc phthalocyanine (ZnPc) in colon cancer by their stabilization in purified, naturally secreted extracellular vesicles (EVs). The PS ZnPc was incorporated in EVs (EV-ZnPc) by a direct incubation strategy that did not affect size distribution or surface charge. By using co-culture models simulating a tumor microenvironment, we determined the preferential uptake of EV-ZnPc toward colon cancer cells when compared with macrophages and dendritic cells. We observed that PDT promoted total tumor cell death in normal and immune cells, but showed selectivity against cancer cells in co-culture models. In vivo assays showed that after a single intravenous or intratumoral injection, EV-ZnPc were able to target the tumor cells and strongly reduce tumor growth over 15 days. These data expose opportunities to enhance the potential and efficacy of PDT using simple non-synthetic strategies that might facilitate translation into clinical practice.

## 1. Introduction

PS are interesting therapeutic agents for cancer therapy as they have the ability to produce reactive oxygen species (ROS) upon external light irradiation, which can be used to locally destroy tumors in a process described as photodynamic therapy (PDT) [1]. PS combined with new technologies, such as improved endoscopic devices, are allowing an increased therapeutic efficacy, by enabling the irradiation of deep-seated tumors and producing ROS in small areas, thus greatly reducing side effects of conventional tumor therapeutics e.g., chemotherapy. Zinc phthalocyanine (ZnPc) is a promising photosensitizer for cancer therapy because of its minimal dark toxicity, excellent singlet oxygen quantum yield, and absorption within the therapeutic window that allows a favorable tissue penetration of the light used for photodynamic effect. Unfortunately, the applicability of this compound is limited due to its low solubility and poor selectivity toward cancer cells [2]. 

Several authors have developed strategies using nanotechnology to encapsulate lipophilic PS and increase their efficacy in different cancer models. As with many others drug delivery strategies, multiple synthetic procedures are needed for encapsulation of the drug, stabilization, and targeting of the nanoparticle to the tumors. From a clinical and industrial point of view, these multiple processes reduce the reproducibility and increase the cost of developing the system, reducing the possibilities of funding for clinical trials. Therefore, the development of simple and synergic strategies to target PS toward cancer cells might have a higher impact and faster track toward clinical applications.

Extracellular vesicles (EVs) are natural particles released from cells delimited by a lipid bilayer which cannot replicate [3,4]. EVs have a great potential for immunotherapy and drug delivery due to their exceptional properties and versatility, which have led to their evaluation in multiple clinical trials [5,6]. As their membrane is equipped with several adhesion molecules, their cellular internalization can be promoted through different processes, such as ligand-receptor interaction, pinocytosis, phagocytosis, or fusion with the plasma membrane [7]. Moreover, nanosized EVs have also passive accumulation in tumors with high vascularization and low lymphatic drainage due to the enhanced permeability and retention (EPR) effect and has been suggested as interesting agents for the delivery of photodynamic agents [8,9,10,11].

Several authors have shown that the cellular origin of EVs has an important effect in their accumulation and in in vivo distribution [12]. For instance, endothelial EVs originated from the brain can effectively cross the blood brain barrier and accumulate in cerebral tissue, and mesenchymal stem cells EVs have shown increased uptake by their own cells when compared with other immune cells [13,14]. In a previous work, we studied the potential of tumor extracellular vesicles (TEVs) derived from melanoma B16F10 cells as a trojan horse strategy to increase selectivity in metastasis [15]. Our results indicated that melanoma EVs had a preferential accumulation in tumor cells when compared with immune cells, fibroblast, and endothelial cells. Interestingly, B16F10 EVs had similar accumulation in colon cancer and melanoma cells, indicating their potential to target other types of cancers than their cell of origin [15]. Although this cell type-related tropism has great potential, it is important to consider that more evidence is required to support this theory. To the best of our knowledge, the cell-type dependent uptake of TEVs in cancer cells has only been addressed in regular culture models. It has been reported that in the tumor microenvironment (TME), cancer cells can modulate other immune cells to avoid immune clearance and promote therapy resistance. Therefore, using co-culture models could provide more representative information about the effectiveness of TEVs for cancer therapy. 

In this study, we developed a synergic strategy to increase efficacy and selectivity of PDT toward cancer cells and tumors. To this end, we evaluated the optimal parameters to load B16F10 melanoma-derived EVs with ZnPc (EV-ZnPc) without affecting size distribution and surface charge. We used co-culture models of colon cancer cells, macrophages, and dendritic cells to stablish their preferential accumulation in tumor cells in regular or complex microenvironments. We performed cytotoxicity assays using PDT and observed that EV-ZnPc was more effective than ZnPc alone, resulting in complete cell death of cancer cells in normal and co-culture models. In addition, EV-ZnPc can effectively reach colon cancer tumors and strongly inhibit tumor growth after either intravenous or intratumoral injection. Our data indicate that EV-ZnPc can potentially be used to simultaneously image, increase selectivity, and locally treat tumors without exerting adverse effects. As the system is easy to produce, it could reduce production costs and facilitate scaling up processes. Moreover, this proof of concept can provide important knowledge and spark ideas about the potential of non-synthetic strategies for cancer theranostics. 

## 2. Materials and Methods

### 2.1. Cell Culture Conditions

Murine macrophages (RAW 264.7), metastatic murine melanoma cells (B16F10), and colon carcinoma cell lines (MC-38) were cultured in Dulbecco’s modified Eagle’s medium with GlutaMAX, Gibco, ref# 31966-021), supplemented with 10% fetal bovine serum and antibiotics (1% penicillin and streptomycin) at 37 °C, 5% CO_2_. Dendritic cells were cultured as described previously [16], in culture medium consisting of Iscove’s modified Dulbecco’s medium (IMDM, Lonza, Basel, Switzerland) supplemented with 8% fetal calf serum (Greiner, Kremsmünster, Austria), 100 IU/mL penicillin/streptomycin (Gibco), 2 mM glutamine (Gibco, Landsmeer, The Netherlands), and 25 μM β-mercaptoethanol (Sigma-Aldrich). Cells were kept in an incubator (Panasonic) at 37 °C and 5% CO_2_.

### 2.2. Preparation of EV-ZnPc

B16F10 cells were grown in 175 cm^2^ flasks under normal cell culture conditions. After reaching 80–90% confluency, the medium was washed three times with PBS and replaced with serum-free media. After 48 h of additional incubation, the supernatant was collected and centrifuged at 300× *g* for 10 min, followed by 2000× *g* for 20 min and 16,000× *g* for 30 min to remove cells and debris. The resulting supernatant was then incubated using an EV-precipitation buffer (Cellsg) and incubated for 1 h at 4 °C. EVs were then precipitated by centrifugation at 16,000× *g* for 60 min. The resulting pellet was resuspended in PBS and incubated with 9 M of ZnPc dissolved in DMSO in a ratio of 9:1 for 1 h at 4 °C. The sample was then placed on top of a size exclusion column (exospin^®^, Cellgs, Cambridge, UK) for purification and centrifuged at 50× *g* for 1 min. Total of 200 uL of PBS was placed on top of the column and centrifugated at 50× *g* for 1 min to obtain EV-ZnPc.

### 2.3. Characterization of EV Preparations 

To determine the surface charge of EVs, samples were diluted 400 fold in PBS, loaded on a disposable polycarbonate capillary cell (DTS 1061, Malvern) and analyzed by laser doppler micro-electrophoresis (LDA) using a Zetasizer Nano-ZS (Malvern). To determine the particle concentration and size distribution, samples were diluted in PBS and analyzed with a Nanosight® NS300 (Malvern) using a detection threshold at 3, camera level of 9, and automatic functions for post-acquisition settings. For cryo-EM analysis, samples were applied on a glow-discharged (2 min in 0.2 mbar air using a EMITECH K950X with glow discharger unit) 300 mesh EM grid (Quantifoil R2/2) and were vitrified using an EMGP (Leica, Germany) at room temperature and 100% humidity. Excess sample was removed by blotting once for 1 s with filter paper (Whatman #1). The blotted grids were plunged into liquid ethane (−182 °C). After vitrification the grid was stored in liquid nitrogen until further use. Grids were mounted in a Gatan 626 cryo-holder for cryo-EM imaging. Cryo-EM imaging was performed on a Tecnai 12 electron microscope (FEI Company, the Netherlands) operated at 120 kV. Images were recorded on a 4 k × 4 k Eagle camera (FEI Company, The Netherlands) at 18,000× magnification (pixel size 1.2 nm) between 5 and 10 µm under focus. To determine the total protein content in EV isolates, the MicroBCA protein assay kit (Thermo Fisher) was used. To detect EV proteins 50 μg of total protein from B16F10 cells and B16F10 EVs were lysed with a buffer containing 20 mM HEPES and 0.5 mM phenylmethylsulfonyl fluoride (PMSF), as well as the phosphatase inhibitor ortho-vanadate (50 mM in PBS 1X). Capillary electrophoresis was then performed using Wes® automated Western Blot Testing (ProteinSimple), using 25-capillary cartridges for 12–230 kDa protein separation (SM-W004) according to the manufacturer’s instructions. Briefly, 0.8 µg/µL of lysed proteins were mixed with the provided SDS/DTT mix, boiled at 95 °C for 5 min and loaded into a microwell plate. Primary antibodies (rabbit) corresponding to anti-Grp94 (Sigma, 1:10), anti-Flotillin-1 (Cell signal, 1:10), anti-HSP70 (Cell signal, 1:100), anti-α6-integrin (Cell signal 1:10), anti-Lamp-1 (Cell signal, 1:10), and anti-β-actin (BioLegend, 1:50) were diluted in the provided antibody diluent and loaded into the plate. Subsequently, blocking buffer, luminol/peroxidase, HRP streptavidin, and secondary anti-rabbit antibodies provided by the manufacturers were loaded, spined for 5 min at 300× *g* and placed into the instrument for the chemiluminescent assay. Data were analyzed using Compass software (ProteinSimple). To evaluate the concentration of ZnPc in EVs, samples were lysed, diluted in DMSO, and measured using a UV-visible spectrophotometry (SpectraMax ID3 microplate reader, Molecular Devices). A calibration curve was performed with ZnPc alone and the extrapolated concentration was used to determine the encapsulation efficiency (EE) using the formula: EE% = amount of ZnPc in EVs/amount of ZnPc added.

### 2.4. Uptake of EVs

D1DCs, MC38, and RAW cells were plated in 24-well plates at a density of 40,000 cells per well for MC38 and 120,000 for RAW cells or D1DCs due to a slower growth rate. Cells were allowed to grow for 18 h and then incubated with 4 µM of either ZnPc alone or EV-ZnPc for additional 24 h. Samples were then collected, washed in PBS, reconstituted in FACS buffer (PBS with 0.5% BSA and 0.02% sodium azide) and kept at 4 °C before analysis by flow cytometry on an LSR II (BD Biosciences). For co-culture studies, MC38 cells were lentivirally transduced with cyan fluorescent protein (CFP) and sorted on a BD FACSARIA II based on CFP+ to obtain MC38-CFP. MC38-CFP cells were seeded in 24-well plates to a density of 40,000 cells with 120,000 RAW cells or D1DCs. Cells were allowed to grow for 18 h, incubated with EV-ZnPc for additional 4 h and harvested as described above. For flow cytometry analysis samples were stained with CD11b-PE (for RAW cells) (1:3200, clone M1/70, BD Biosciences) or CD11c-PE (for D1DCs) (1:200, clone N418, Biolegend), 0.5 µM 4′,6-diamidino-2-phenylindole (DAPI) (Sigma-Aldrich) in FACS buffer prior to analysis on an LSR II (BD Biosciences).

### 2.5. Cell Viability and PDT In Vitro

For PDT studies, MC38 cells were plated in 24-well plates at a density of 40,000 cells per well and allowed to grow for 18 h prior incubation with 4 µM of either ZnPc alone or EV-ZnPc for additional 24 h. Samples were then collected as described above prior to analysis by flow cytometry using Annexin V-FITC (BD Pharmingen) and 0.5 µM DAPI staining. To evaluate the effect on immune cells, D1DCs, RAW, and MC38-CFP cells were seeded in 24-well plates to a density of 40,000 MC38-CFP cells and 120,000 D1DCs and RAW cells at 37 °C, 5% CO_2_. Cells were then incubated with EV-ZnPc for 24 h, washed with PBS and incubated with fresh medium prior to illumination with a 662 nm Milon Lakhta Laser. The distance between sample and laser (~10 cm) as well as the laser power were adjusted to reach a fluence rate of 100 mW/cm^2^ for a total light dose (fluence) of 20 J/cm^2^. After PDT, cells were incubated for an additional 24 h, stained with CD11b-PE (for RAW cells) or CD11c-PE (for D1DCs), as well as with Annexin V-FITC (BD Pharmingen) and 0.5 µM DAPI before analysis on an LSR II flow cytometry.

For the co-culture analysis, MC38-CFP cells were seeded to a density of 40,000 cells in 24-well plates with 120,000 of either D1DCs or RAW cells. Cells were then incubated with EV-ZnPc for 24 h, washed with PBS and incubated with fresh medium prior to light exposure as described above. After PDT cells were incubated for additional 24 h, stained with DAPI and a mixture of CD11b-FITC and CD11c-FITC, and analyzed by flow cytometry.

### 2.6. Animal Experiments

All animal experiments were conducted in accordance with the Code of Practice of the Dutch Animal Ethical Commission. Female C57BL/6J mice of 6–8 weeks old were purchased from ENVIGO (Horst, the Netherlands), C57BL/6-albino mice were bred in the breeding facility of the Leiden University Medical Center (LUMC) and used for experiments between the ages of 6 and 12 weeks. All mice were kept under specified pathogen-free conditions and housed in polycarbonate cages placed in a ventilated, temperature-controlled room at 20 °C and 10% relative humidity, under a 12-h light/dark cycle and were acclimatized to this environment for at least one week prior to treatment.

### 2.7. Distribution and In Vivo PDT

C57BL/6-albino mice were inoculated with 0.5 × 10^6^ MC38 tumor cells in 200 μL PBS on the right flank and randomly divided into groups when tumors reached a size of approximately 125 mm^3^. Mice were then injected with either EV-ZnPc (400 µM) or EV alone in 100 µL PBS for intravenous (IV) administration into the tail vein or in 30 µL for intratumoral (IT) administration. The concentration of EV alone was administrated according to the amount of protein content in the 400 µM EV-ZnPc. Non-invasive fluorescence imaging was performed at 0, 2, 6, 24, 50, and 70 h using the IVIS Spectrum (Perkinelmer) under isoflurane anesthesia. Relevant areas were shaved right before measurement to minimize interference of the fluorescent signal. For PDT, C57BL/6J mice were inoculated with 5 × 10^6^ MC38 tumor cells in 200 μL PBS on the right flank and randomly divided into groups when tumors reached a size of approximately 125 mm^3^. Mice were then injected with EV-ZnPc and illuminated with 662 nm light under isoflurane anesthesia at a DLI of 6, 24, and 48 h after EV administration. Laser power and distance between mice and laser (~10 cm) were adjusted to reach a fluence rate of 116 mW/cm^2^ over 1000 s for a fluence of 116 J/cm^2^ . Mouse conditions, weight, and tumor sizes were measured regularly for the entire duration of the experiment.

### 2.8. Statistical Analysis 

Statistical analyses of the data were performed using Prism 8.0 (GraphPad Software Inc. San Diego, CA, US) by applying the nonparametric test ANOVA followed by the Tukey post-test for all *p*-values unless specified in the text. All results are expressed as mean ± SEM.

## 3. Results and Discussion

### 3.1. Preparation and Characterization of EV-ZnPc

**Figure 1:**
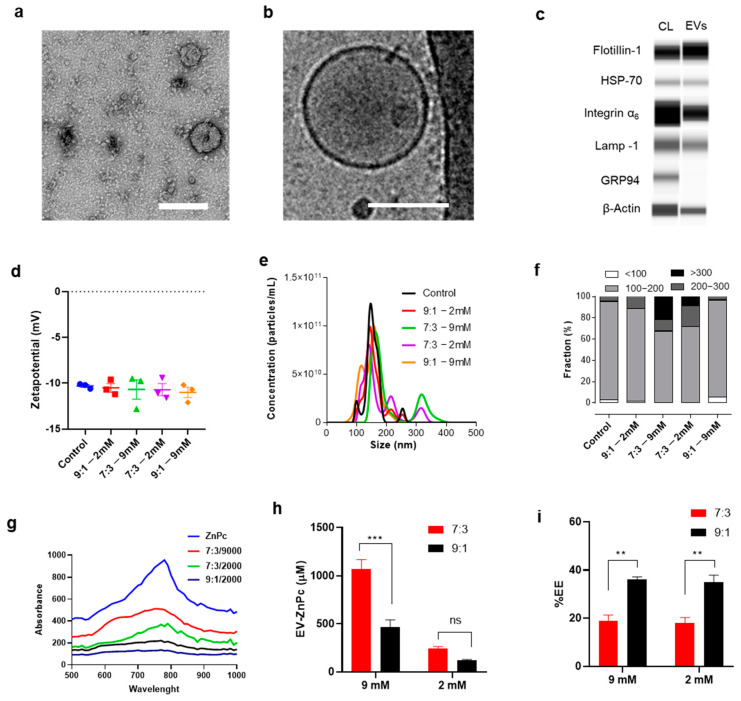
Preparation of EV-ZnPc. Representative (**a**) TEM, (**b**) cryo-TEM micrograph, and (**c**) capillary electrophoresis (CL = cell lysates) of EVs isolated from B16F10. EVs were loaded with different concentrations of ZnPc and characterized by (**d**) laser doppler anemometry and (**e**) NTA. (**f**) The total number of EVs obtained by NTA was used to calculate the proportion of different size fractions. UV-vis spectroscopy was used to obtain the (**g**) absorption curves, (**h**) concentration of samples, and (**i**) encapsulation efficiency (EE). Scale bars are 100 nm. *** *p* < 0.001; ** *p* < 0.01; mean ± SEM; *n* = 3).

Naturally targeting EVs were obtained from the culture media of melanoma B16F10 cells using a previously established protocol [15] that resulted in EVs with a typical vesicular shape and size close to 120 nm (Figure 1a,b). By capillary electrophoresis we analyzed the presence of transmembrane and cytosolic proteins commonly associated with EVs. Integrin α1, Lamp-1, Flotillin-1, HSP70, and β-actin were present in both EVs and cell lysates while the endoplasmic reticulum marker GRP94 (negative control) was only detected in the cell lysates (Figure 1c). 

ZnPc was incorporated in the EVs following a direct incubation method, which is a straight-forward technique to incorporate drugs in EVs as it does not require any additional reagent or synthetic step [8]. Although the incubation method is simple, it remains challenging to obtain a high concentration of lipophilic compounds without affecting vesicular distribution as such drugs have to be dissolved in membrane-disrupting solvents such as ethanol or DMSO. To address this issue, we incubated the EVs with two different concentrations of ZnPc (2 mM and 9 mM) and using two different EV/DMSO proportions (9:1 and 7:3). These concentrations were chosen to compare the loading efficiency of a saturated (9 mM) vs. non-saturated ZnPc solution (2 mM) and to evaluate the effect of high DMSO proportions (7:3) vs. a previously validated one (9:1) [17]. The resulting vesicles showed a zeta potential of -11 mV and a NTA median size close of 130 nm for all the concentrations/proportions tested (Figure 1d,e). A further exploration on the size distribution revealed that most of the vesicles were between 100 and 200 nm and that by using 9:1 proportions of EV/DMSO the size distribution did not change regardless of the amount of ZnPc used. At 7:3 proportions, however, we observed an increase in the number of particles >200 nm, which could be due to DMSO-related disruption/aggregation of EVs (Figure 1f). By using UV-vis spectroscopy we were able to obtain the spectra of the samples and an estimation of the concentration and encapsulation efficiency (Figure 1g–i). As expected, using a higher proportion of ZnPc/DMSO (7:3) resulted in a higher association of ZnPc in EVs, but also resulted in significatively lower encapsulation efficiency, independent of the concentration of ZnPc used (Figure 1h,i). These results suggest that the most efficient way to obtain a high amount of a lipophilic compound encapsulated in EVs is to use the highest concentration of the compound possible in the lowest volume of solvent possible. In this case, 9 mM was the highest concentration possible for ZnPc dissolved in DMSO without saturating the solution, thereby for further analysis samples, stocks solutions were made with 9 mM of ZnPc at 9:1 proportions.

### 3.2. Tumor Preferential Uptake of B16F10-EVs

**Figure 2:**
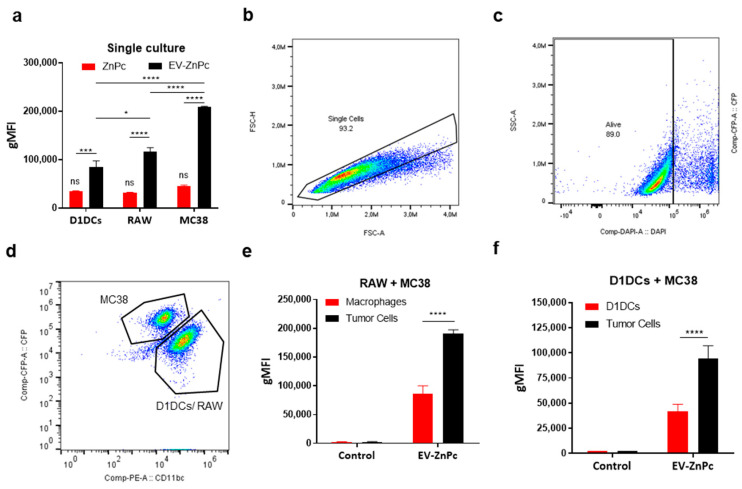
Cell type dependent uptake of EV-ZnPc. (**a**) MC38, D1DCs, or RAW cells were treated with ZnPC alone or EV-ZnPc for 24 h prior to fluorescent detection by flow cytometry using Annexin-V/DAPI staining. MC38-CFP cells were co-cultured with either D1DCs or RAW cells and incubated with EV-ZnPc as described in methods. The fluorescence in the subpopulations was detected by flow cytometry. Gating was performed to exclude debris (not shown), (**b**) single cells and (**c**) dead cells. (**d**) CFP and CD11b/c were used to distinguish the uptake between (**e**) tumor cells and D1DCs or (**f**) tumor cells and macrophages. Data are means ± SEM (*n* = 3). **** *p* < 0.0001; *** *p* < 0.001; * *p* < 0.05.

To determine if EV-ZnPc can promote PS delivery toward colon cancer cells preferentially, we compared the fluorescence in MC38 cells, D1 dendritic cells (D1DCs), and RAW cells after incubation with either ZnPc alone or EV-ZnPc over 24 h. By flow cytometry analysis, we observed that the fluorescent signal of EV-ZnPc was higher than non-formulated ZnPc in all the cells of study and that MC38 cells presented the highest signal of EV-ZnPc compared to D1DCs and RAW cells (Figure 2a). These results correspond to our observations from previous work indicating that B16F10 EVs are preferentially taken up by other tumor cells and can be used to increase drug delivery of therapeutic compounds [15]. As in real conditions tumor cells are not independent cells but rather coexist in a microenvironment that can induce changes in their phenotype and in their response, we sought to evaluate if the preferential uptake was maintained in co-culture conditions of tumor cells with macrophages and D1DCs. Therefore, we cultured RAW cells + cyan fluorescent protein (CFP) expressing MC38 cells (MC38-CFP) or D1DCs + MC38-CFP cells. Separation of tumor cells versus RAW or D1DCs was based on CFP versus CD11b or CD11c (CD11b/c), respectively, allowing differentiation between tumor and immune cells (Figure 2b–d). As shown in Figure 2e,f, the fluorescent signal of EV-ZnPc was significatively higher in MC38 cells in both cultures of MC38/RAW or MC38/D1DCs, indicating that the preferential uptake was maintained in co-culture models. These results support the hypothesis that tumor-derived EVs can preferentially select cancer cells over other immune cells and provide novel evidence on the uptake of EVs in more complex models simulating a TME. Using TEVs as trojan horses might be an interesting strategy to increase the delivery of drugs toward resistant tumors and reduce the side effects of therapies by reducing off-target effects. EV internalization and cargo-delivery is still a subject of study and debate as it has shown to be extremely varied involving passive fusion process as well as multiple clathrin-dependent and clathrin-independent endocytic pathways [18]. After internalization EVs can be localized within double membrane structures and colocalizing with various endosomal, lysosomal, phagocytic and lipid raft markers on recipient cells [19]. Although the mechanisms of enhanced uptake of TEVs on specific cells are not totally understood, it has been suggested that adhesion molecules play an important role in the process. For example, surface heparan proteoglycans have been involved in increased uptake by cancer cells [20] while intercellular adhesion molecules (ICAMs) can increase uptake by immune cells [21]. Proteomic analysis of B16F10 and other melanoma-derived EVs have indicated a high expression of proteoglycan proteins [22], which can explain their enhanced uptake by other tumor cells as observed in these results. 

### 3.3. Effect of PDT In Vitro

**Figure 3:**
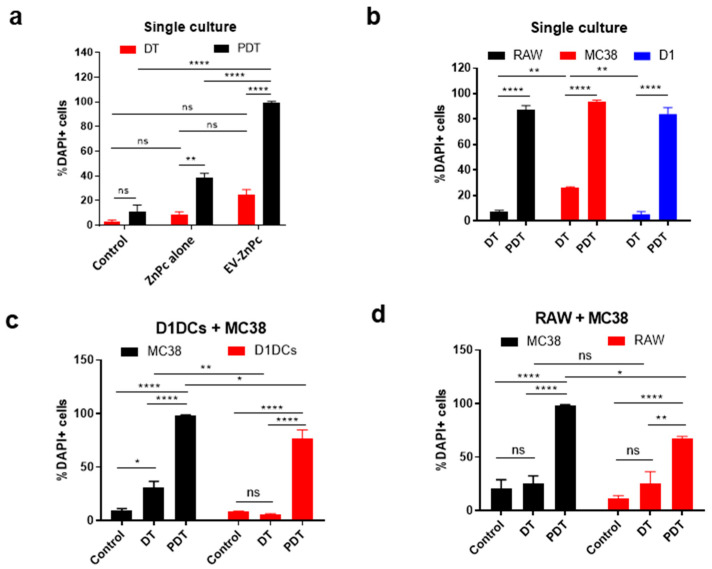
EV-ZnPc effect on normal and tumor cells after PDT in vitro. (**a**) MC38 cells were incubated with ZnPc alone or EV-ZnPc cells prior to evaluation of dark toxicity (DT) and PDT-induced cytotoxicity by flow cytometry using Annexin-V/DAPI staining. PDT efficacy was evaluated in RAW, MC38, and D1DCs cells in (**b**) normal culture models and co-cultures of (**c**) D1DCs + MC38 and (**d**) RAW + MC38 cells. CFP and CD11b/c were used to distinguish the uptake between tumor cells and D1DCs or tumor cells and macrophages. Data are means ± SEM (*n* = 3). **** *p* < 0.0001; ** *p* < 0.01; * *p* < 0.05.

We evaluated the dark toxicity and toxicity after PDT using a Annexin-V/DAPI staining to address the effect on early/late apoptosis. When cells were not exposed to light irradiation, ZnPc alone promoted cell death of ~8% of cells while EV-ZnPc resulted in the death of ~20% of the cells suggesting an effect of EV-ZnPc alone on the apoptosis of cancer cells (Figure 3a). Although these differences were not significant, TEVs have previously been shown to promote an apoptotic effect in other cancer cell lines by increasing Bax and decreasing Bcl_2_ expression, which could explain this increase [23]. For PDT assays, we illuminated the cells with 662 nm light, 24 h prior to DAPI/Annexin V staining. Interestingly, PDT treatment of MC38 cells incubated with EV-ZnPc resulted in cell death of over 99% of cells while treatment with ZnPc alone was at least two times less effective, indicating that EVs can increase the efficacy of ZnPc for PDT (Figure 3b). ZnPc has been described as an excellent photodynamic agent due to its ability to generate cytotoxic single oxygen species, promoting apoptosis, ER stress, necrosis, and autophagy [24]. As EV-ZnPc did not show significant effects without illumination, the enhanced effect after PDT is likely related to ZnPc stabilization and favored delivery after vectorization. ZnPc photochemical activity relies on his monomeric form and therefore his efficacy has shown to decrease after aggregation [24]. For this reason, other nanosized delivery strategies have also been employed by other authors to increase their efficacy such as liposomes and polymeric micelles [25,26]. To determine the effect of PDT in a co-culture model, we used a similar gating approach as before to differentiate between tumor and immune cells. Interestingly, in a single cell culture condition, the dark toxicity of EV-ZnPc in macrophages and D1DCs was significatively lower than MC38 cells, while the effect of PDT was similar between MC38, D1DCs, and RAW cells, resulting in more than 85% cell death for all the groups (Figure 3d). In co-culture conditions however, the data show that dark toxicity of EV-ZnPc only had an effect on MC38 cells that were cultured with D1DCs (Figure 3c,d) and the effect of PDT was higher on MC38 cells in cultures of D1DCs/MC38 cells and RAW/MC38 cells. Although we only observed a slight difference (*p* < 0.05) in the selectivity of PDT between cancer and immune cells under co-culture conditions, it is important to note that in all the cases PDT promoted 100% death of all tumor cells. As PDT was highly effective overall, using lower concentrations of EV-ZnPc could increase the gap between the groups without compromising efficacy on cancer cells. 

### 3.4. Distribution and PDT In Vivo

**Figure 4:**
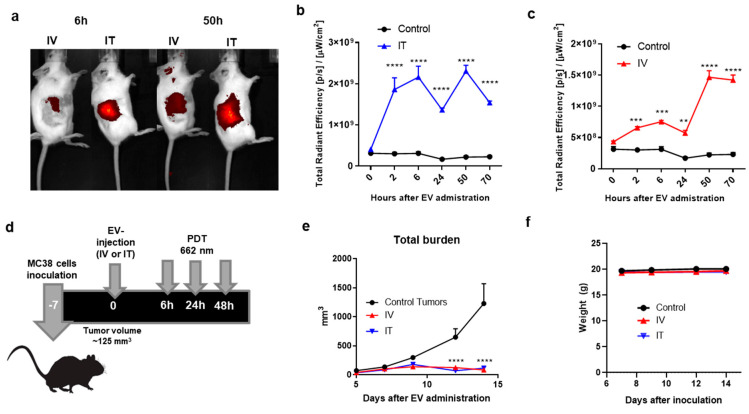
Biodistribution and effect of EV-ZnPc after PDT in vivo. Mice were inoculated subcutaneously with 0.5 × 10^6^ MC38 cells in 200 μL PBS in the right flank. At day 7, when the tumors were an average size of ~125 mm^3^, EV-ZnPc were injected intravenously (IV) or intratumorally (IT). Non-invasive fluorescence imaging was performed at 2, 6, 24, 50, and 70 h after injection using the IVIS fluorescence spectrometer. (**a**) Representative NIR700 fluorescence imaging in the tumor area of mice at 6 and 50 h after injection. Fluorescence intensity of the NIR800 signal after (**b**) IT or (**c**) IV injection. (**d**) Schematic representation of protocol used for PDT assay: mice were inoculated with MC38 cells in the flank and injected with EV-ZnPc as mentioned above. PDT was performed by illumination of the tumor on the right flank with 662 nm light at 116 mW/cm^2^ for 116 J/cm^2^ over 1000 s. (**e**) Tumor growth curves and (**f**) weight of the animals were evaluated over 14 days after inoculation. Data are means ± SEM from *n* = 4 mice per condition. **** *p* < 0.0001; *** *p* < 0.001; ** *p* < 0.01.

To explore the potential of EV-ZnPc in animal models, we compared the accumulation of EV-ZnPc in MC38 tumors after either intravenous (IV) or intratumoral (IT) administration. C57BL6 tumor-bearing mice were injected with EV-ZnPc (IT or IV) and non-invasively imaged over 70 h. As shown in Figure 4a,b, following IT administration we immediately observed a fluorescent peak in the tumor area which was sustained over 70 h. After IV administration the fluorescence in the tumor increased over time, reaching a peak that is comparable to the one observed in IT administration after 50 h (Figure 4a–c). Even though IT administration results in a higher and more constant accumulation overtime, it is important to consider that IV administration is a more realistic administration route as most of the tumors in humans are not visible as in subcutaneous animal models of cancer. Our results suggest that EV-ZnPc can effectively target and be retained in MC38 tumors after either IT or IV administration. The high tumor accumulation observed after IV administration might be related to a combination of the EPR effect and the enhanced uptake due to the multiple adhesion molecules. For PDT, tumor-bearing mice were treated with EVs and irradiated with a 662 nm laser three times, as illustrated in Figure 4d. As expected, untreated tumors grew out rapidly, reaching humane endpoints at around 20 days post inoculation. Mice treated with a single IT or IV administration of EV-ZnPc, displayed a strongly inhibited tumor growth for 16 days after treatment. Moreover, we did not observe changes in mouse weight, which indicates that there was no treatment-induced toxicity. 

Overall, our data suggest that EV-ZnPc can effectively target colon cancer tumors and promote tumor growth inhibition after PDT. It is important to mention that ZnPc alone was not administrated in mice, as it had to be dissolved in DMSO prior to administration which is a harmful solvent for animals. Due to the low solubility of ZnPc, other reports have indicated that his administration lead to crystallization, low target specificity, and strong liver accumulation resulting in decreased efficacy [27,28]. As shown in this work, using EVs could be an interesting strategy to increase stability of ZnPc and enhance its potential for cancer therapy. It has previously been shown that EVs can outperform other drug delivery systems including liposomal formulations of other photodynamic agents with low solubility [9,10,11]. The natural selectivity of B16F10-EVs can potentially be a cost-effective way to deliver PS to tumors systemically. By performing a direct incubation step without surface functionalization, it was possible to simultaneously target, image, and reduce tumor growth in colon cancer tumors which is a significant advantage compared to other methods [6,12,15]. Although the tumors in this study were targetable for laser illumination, in previous work, we also showed that B16F10 EVs can effectively reach metastatic tumors which are usually hard to reach as they do not depend on the EPR effect [15]. Despite the fact that this is a promising therapy, there are still several challenges for deep tissue PDT, as even the most promising PS are limited to only few centimeters of light penetration through the skin.

It is also important to consider that the utilization of TEVs is still controversial as a therapy, as reports indicate their role in tumor growth and preparation of the metastatic niche for metastasis [29]. These effects were, however, observed after multiple administration of EVs as opposed to our study methodology. Moreover, our data indicate that EV-ZnPc without laser illumination promoted a slight increase in the apoptosis of colon cancer cells. Some studies have also used EVs derived from tumor cells to activate immune cells, as they present tumor antigens (TAAs) that may induce tumor-specific immune responses [30]. As PDT-induced apoptosis leads to the release of DAMPs and TAAs, the combination of tumor EVs and PDT can act like a synergic therapy to boost the immune system and prevent tumor recurrence. In the future we might be able to remove the negative effects of such EVs by for example inactivating their genetic material, while maintaining their surface targeting proteins and antigens. These would potentially allow to have all the benefits of TEVs without their potential risk of cancer promotion. 

## 4. Conclusions

In this work, we report on the development, characterization, and evaluation of EV-ZnPc for the treatment of colon cancer tumors. We developed a straightforward strategy to exploit the potential of a PS with low solubility (ZnPc) by direct incubation and size exclusion separation. Using co-culture studies simulating a tumor microenvironment, we observed that EV-ZnPc was preferentially taken up by MC38 cells when compared to D1DCs and RAW cells. We determined that EV-ZnPc slightly promoted the apoptosis of cancer cells but not of D1DCs or RAW cells without light irradiation. After light exposure in a regular culture model, PDT promoted total tumor cell death in normal and immune cells, but showed selectivity against cancer cells in co-culture models. In an animal model of colon cancer, we were able to observe the accumulation of EV-ZnPc in tumors after either intravenous or intratumorally injection and determine their effect after PDT which resulted in a strong tumor growth inhibition over 16 days after either IV or IT administration. Overall, these data provide valuable information about the potential of EV-ZnPc to increase efficacy and selectivity toward cancer-specific cells that could be used for the development of simpler theranostic strategies with a more reliable clinical translation.

## Data Availability

Data sharing not applicable.
